# Supplementary material for: Suitability of native milkweed (Asclepias) species versus cultivars for supporting monarch butterflies and bees in urban gardens
Source: PeerJ. 2020 Sep 25;8:e9823. doi: 10.7717/peerj.9823 (PMC7521339; doi:10.7717/peerj.9823)
Supplement: Supplemental Information 1 [file peerj-08-9823-s001.docx]

| **Table S1. Ornamental characteristics of wild-type milkweeds and cultivars** | | | | |
| --- | --- | --- | --- | --- |
| Milkweeds | Bloom color | Foliage color | Means of production^1^ | Additional features^5^ |
| *A. incarnata* |  |  |  |  |
| Wild Type | pink | green | OP^2^, seeds | ----- |
| Cinderella | dark pink | green | CP^3^, seeds | larger flower clusters |
| Ice ballet | white | pale green | CP, seeds | shorter stature |
| Soulmate | pink | green | CP, seeds | more flower clusters |
| *A. tuberosa* |  |  |  |  |
| Wild Type | orange | dark green | OP, seeds | ----- |
| Blonde Bombshell | pale yellow | green | CR^4^, tissue culture | clonal |
| Gay Butterflies | red, orange, yellow | green | CP, seeds | multiple bloom colors^6^ |
| Hello Yellow | bright yellow | green | CP, seeds | larger stature |
| ^1^Information from commercial sources and Dr. R. Geneve pers. comm. | | | | |
| ^2^OP, Open pollination: Plants that are naturally pollinated by insects in an open field setting | | | | |
| ^3^CP, Controlled pollination: Mechanical pollination from known parental plants and exclusion of outside pollen sources | | | | |
| ^4^CR, Clonal reproduction: Vegetative reproduction from a parent plant | | | | |
| ^5^Visual observations compared to Wild Type | | | | |
| ^6^ Plants in our experiment expressed all three bloom colors (within plant, but not within umbel) in varying degrees. | | | | |
