# Supplementary material for: Suitability of native milkweed (Asclepias) species versus cultivars for supporting monarch butterflies and bees in urban gardens
Source: PeerJ. 2020 Sep 25;8:e9823. doi: 10.7717/peerj.9823 (PMC7521339; doi:10.7717/peerj.9823)
Supplement: Supplemental Information 2 — *denotes significant difference compared to wild-type within species, ANOVA, 2-tailed Dunnett’s test, P < 0.001 [file peerj-08-9823-s002.docx]

| **Table S2. Height and width at maturity of milkweeds**  **in the replicated gardens in 2019**^1^ | | | |
| --- | --- | --- | --- |
|  | Mean height (cm) | Mean canopy width (cm) |  |
| ***A. incarnata*** |  |  |  |
| Wild type | 89 ± 5.3 | 68.4 ± 5.6 |  |
| ‘Cinderella’ | 91 ± 5.6 | 77.9 ± 3.2 |  |
| ‘Ice Ballet’ | 77.5 ± 4.2 | 77.8 ± 6.1 |  |
| ‘Soulmate’ | 99.3 ± 1.7 | 95.3 ± 4.8* |  |
|  |  |  |  |
| ***A. tuberosa***^2^ |  |  |  |
| Wild type | 32.5 ± 0.7 | 36.6 ± 1.1 |  |
| ‘Gay Butterflies’ | 48.1 ± 2.3* | 58.3 ± 2.4* |  |
| ‘Hello Yellow’ | 45.6 ± 1.9* | 51.1 ± 2.6* |  |
| ^1^ANOVA (all cultivars): *F*_6,30_ = 73.0, 23.9 for mean height and canopy width, respectively; both *P* < 0.001.  * denotes significant within-species difference compared to wild-type; 2-tailed Dunnett’s test, *P* < 0.001.  ^2^cultivar 'Blonde Bombshell' was excluded due to poor regeneration in the gardens in 2019 | | | |
|  | | | |
